# Validation of telesimulation in the care of late preterm newborns with hypoglycemia for nursing students

**DOI:** 10.1590/0034-7167-2022-0438

**Published:** 2023-12-08

**Authors:** Débora Schimitt Porto, Maria Luzia Chollopetz da Cunha

**Affiliations:** I Universidade Federal do Rio Grande do Sul. Porto Alegre, Rio Grande do Sul, Brazil

**Keywords:** Validation Study, Premature Newborn, Simulation Training, Telesimulation, Nursing Students, Estudio de Validación, Recién Nacido Prematuro, Entrenamiento de Simulación, Telesimulación, Estudiantes de Enfermeira, Estudo de Validação, Recém-Nascido Prematuro, Treinamento Por Simulação, Telessimulação, Estudantes de Enfermagem

## Abstract

**Objective:**

To develop and validate a telesimulation scenario for nursing students in the care of late preterm infants with hypoglycemia.

**Methods:**

A methodological study conducted between August 2021 and May 2022 in a virtual environment involved constructing and validating the scenario with 10 experts, and testing it with 10 students. The content validity index assessed validity, with a threshold of 80% or higher, and suggestions were analyzed using semantic approximation.

**Results:**

Validation confirmed the appropriateness of all 14 scenario items, with an overall index of 97.8% and clarity and relevance indices of 98.5%. During testing, the overall index was 99.7%, with the “resources” item receiving the lowest score. Adjustments were made to objectives, technical terms, resources, and target audience based on feedback.

**Conclusion:**

Telesimulation is a widely accepted educational technology for training nursing students, with potential to enhance teaching quality and neonatal care.

## RESUMO

**Objetivo:**

Elaborar e validar um cenário de telessimulação para estudantes de enfermagem no cuidado ao prematuro tardio com hipoglicemia.

**Métodos:**

Estudo metodológico, realizado entre agosto de 2021 e maio de 2022, em ambiente virtual envolvendo construção e validação de conteúdo com 10 especialistas e teste do cenário com 10 estudantes. Foi utilizado índice de validade de conteúdo igual ou superior a 80% e análise de sugestões por aproximação semântica.

**Resultados:**

Na validação, os 14 itens do cenário foram considerados adequados, com índice geral de 97,8% e 98,5% para clareza e pertinência, respectivamente. No teste do cenário, o índice foi de 99,7%, sendo que o item “recursos” obteve o menor escore. Foram realizados ajustes nos objetivos, termos técnicos, recursos e público-alvo.

**Conclusão:**

A telessimulação é uma tecnologia educacional viável e aceita na capacitação de estudantes de enfermagem e com potencial para melhorar a qualidade do ensino e da assistência neonatal.

## RESUMEN

**Objetivo:**

Desarrollar y validar un escenario de telesimulación para estudiantes de enfermería en el cuidado de recién nacidos prematuros tardíos con hipoglucemia.

**Métodos:**

Estudio metodológico virtual entre agosto de 2021 y mayo de 2022, con construcción y validación del escenario por 10 expertos y prueba con 10 estudiantes. Se utilizó un índice de validez de contenido con umbral del 80% o superior y se analizaron sugerencias mediante aproximación semántica.

**Resultados:**

Validación confirmó la adecuación de los 14 elementos del escenario, con un índice general del 97.8% y claridad y pertinencia del 98.5%. En la prueba del escenario, el índice general fue del 99.7%, con “recursos” obteniendo la puntuación más baja. Se ajustaron objetivos, términos técnicos, recursos y público objetivo según comentarios recibidos.

**Conclusión:**

Telesimulación es una tecnología educativa ampliamente aceptada para formación de estudiantes de enfermería, con potencial para mejorar la calidad de la enseñanza y el cuidado neonatal.

## INTRODUCTION

Late preterm newborns (LPTNB), also known as late preterm infants, account for approximately 70% of all births occurring before 37 weeks of gestation. They are classified as such for being between 34 and 36 weeks and 6 days. Due to their physiological immaturity, they are at a higher risk of hypoglycemia, a condition that can lead to admission to intensive care units and prolonged hospitalization^(1, 2)^.

Hypoglycemia is one of the most prevalent complications in this population, attributed to difficulties in establishing breastfeeding and limited glucose reserves compared to full-term newborns^(1, 2)^. Therefore, glucose monitoring using strips is recommended at intervals of 2, 4, 6, 12, 24, 48, and 72 hours of life. Rapid treatment is initiated following the established protocol to prevent brain damage upon identifying hypoglycemia^(2)^. Given the relevance of this issue, there is a need to enhance teaching strategies and interventions focusing on the education of nursing students, as the quality of care for this at-risk group depends on the adequate training of future professionals.

Among the teaching strategies in neonatal care, clinical simulation stands out. It is currently considered a promising tool as it ensures a safe environment for students to develop competencies. Simulation in education can take place face-to-face in teaching laboratories, simulation centers, and hospitals, as well as through remote simulation using new technologies such as virtual reality, serious games, and telesimulation^(3)^.

With the advent of the COVID-19 pandemic caused by the novel coronavirus (SARS-CoV-2) in March 2020 and subsequent institution closures, telesimulation has gained momentum in nursing education. Efforts have been made to adapt laboratory practices to virtual and synchronous activities due to student safety concerns^(4)^. Recently, the International Nursing Association of Clinical and Simulation Learning (INACSL) and the Society for Simulation in Healthcare have expressed support for the use of this technology during the health crisis^(5)^.

Telesimulation is defined as a branch of clinical simulation characterized by the promotion of remotely conducted synchronous practices through video calls. It emerged in the mid-2000s with the aim of facilitating communication between instructors and geographically distant participants with limited access to technological and material resources. Due to the pandemic, there has been a significant increase in the use of this technology, particularly in the past two years. Even with the end of the state of emergency, it has the potential to reduce inequality in access to simulation-based education^(6)^. Research demonstrates the implementation of this modality in the training of physicians and nurses in neonatal and pediatric emergency care^(7, 8)^.

The development of telesimulation relies on planning technological resources and the virtual environment^(^6,9^)^. The construction of the scenario is the first step in any simulation experience, whether in-person or remote. Additionally, content validation by a group of experts in the relevant field is crucial to ensure realism and reproducibility^(10, 11)^.

Given the current growth of telesimulation in the scientific literature, it is worthwhile to produce validation studies of scenarios using this innovative technology in nursing education. Therefore, it is important to clearly describe the approach adopted in the instrument under study, utilizing well-founded theoretical and methodological frameworks. This research can contribute to improving the learning outcomes of students and professionals, as well as the development of clinical competencies relevant to neonatal nursing practices^(12)^.

## OBJECTIVE

To develop and validate a telesimulation scenario for nursing students in the care of late preterm infants with hypoglycemia.

## METHODS

### Ethical aspects

The study was approved by the Research Ethics Committee, following submission to the Plataforma Brasil, in compliance with the guidelines of Resolution No. 466/12 of the National Health Council^(13)^ and Circular Letter No. 2/2021/CONEP/SECNS/MS for research in a digital environment^(14)^. Participants provided informed consent by signing the Informed Consent Form (ICF).

### Study design, period, and location

A methodological study was conducted following the framework proposed by Coluci, Alexandre, and Milani^(15)^, adapted for the development of the telesimulation scenario. The construction and content validation with experts occurred between August and October 2021, while the scenario testing with nursing students from the School of Nursing at the Federal University of Rio Grande do Sul (UFRGS) occurred in April 2022. All stages of the study were conducted in a virtual environment.

### Population, inclusion and exclusion criteria

The criteria for selecting experts in the validation phase of the telesimulation scenario for the care of late preterm infants with hypoglycemia were based on the model proposed by Fehring^(16)^ and adapted by Paula^(17)^. The criteria included: a) doctoral degree with a thesis in the area (5 points); b) master’s degree with a dissertation in the area (4 points); c) specialist degree in the area (3 points); d) publication of articles or abstracts in the area (2 points); e) 1 year or more of clinical experience (1 point); f) awards in the area (1 point). Experts were identified using the advanced search tool in the Lattes Platform of the National Council for Scientific and Technological Development (https://lattes.cnpq.br/) and through professional recommendations. Contact was made via the email provided on the platform.

Regarding the inclusion criteria, participants had to: 1) meet a minimum of 5 points according to the adapted Fehring criteria^(17)^; 2) be a nurse with professional experience and/or education and/or teaching in the maternal-child or neonatology area and/or simulation. The exclusion criteria included: not being available to participate in the research. It is recommended to have between 5 to 10 participants in the expert committee^(15)^. Out of the 60 experts screened, 54 were eligible and invited, 6 were excluded for not meeting the criteria, 10 accepted to participate in the study, and 44 did not respond to the invitation. The selection of experts was based on convenience.

For the scenario testing, participants were selected based on convenience through invitations on social networks and voluntary enrollment. The inclusion criteria were: 1) being a nursing student; 2) having completed or currently taking the nursing care discipline in newborn, child, and adolescent health; 3) being 18 years old or older. The exclusion criteria were: 1) not having technological and communication resources; 2) not being available for synchronous participation. A total of 10 students from the School of Nursing at UFRGS were selected. The literature recommends having between 10 and 40 participants^(18)^.

The study was conducted in three stages: development of the telesimulation scenario, content validation, and scenario testing. The neonatal health care protocol published by the Brazilian Ministry of Health^(2)^ was used for development. The criteria from the INACSL simulation design^(9)^ were followed to organize the elements of the scenario. It was structured into 14 items: telesimulation name, main learning objective, secondary objectives, estimated duration, necessary resources and materials, guidance to actors, participants, prebriefing, scenario description, clinical case description, running, teledebriefing, summary of activity by the instructor, and observers’ checklist.

Subsequently, a pilot of the scenario was conducted with three nursing students from UFRGS, who were randomly selected. They were invited via email and, after accepting, were briefed on the proposal. Reading material was sent in advance, containing the date, time, and link to the virtual room. Experts in simulation, faculty, and clinical nurses from the neonatal field participated in the creation and assessment of the scenario pilot. Students were informed that participation in the pilot would preclude them from participating in subsequent phases of the study.

Content validation took place in two phases: individual and collective. In the individual phase, experts received an invitation letter via email with the telesimulation scenario, the validation form, and, via Google Forms, the Informed Consent Form (ICF) and the characterization instrument, containing the following variables: Gender; Education and Experience in teaching, clinical practice, and research in the neonatal field and/or in simulation/telesimulation

The validation form contained 14 items of the scenario and response options on a 4-point ordinal Likert scale. This was used to evaluate attributes of clarity (not clear, somewhat clear, very clear, extremely clear) and relevance or representativeness (not representative, requires major revision to be representative, requires minor revision to be representative, item is representative). Experts were also prompted to record suggestions in a specific section of the instrument. They were given a 15-day deadline to return their evaluations.

During the collective phase, the committee of experts met with the researcher in a synchronous virtual meeting to discuss suggestions from the validation forms. Nine participants attended the meeting, while one did not. A subsequent meeting was scheduled with the absent expert to conclude this phase. The validated version of the telesimulation scenario was then obtained and sent to each committee member for approval.

The scenario testing occurred in two synchronous videocon-ference sessions using the Microsoft Teams® platform, with four and six students, respectively. Trained operators, actors assigned to play the roles of the mother and the doctor, and a facilitator with education and experience in conducting clinical simulations were invited to participate. All participants had access to a computer or smartphone, microphone, and camera during the case development.

A pre-briefing was conducted using an expository-dialogue lecture strategy. Following the briefing, the running phase commenced with screen sharing, displaying an image of a hypotonic late preterm infant in an incubator, simultaneously with the virtual patient monitor screen (Vital Sign Simulator®) showing no data. This system allowed for changes in the patient’s clinical situation throughout the telesimulation. Only the mother and two participants in the role of nurses were visible on the computer screen. The medical actress turned on the camera only when called to attend the case. Participants analyzed the images, clinical data, and clues, and based on their decision-making, verbalized the necessary actions to resolve the case. In this context, the other students observed the situation with the help of a checklist.

Following the conclusion of the scenario, the teledebriefing began, facilitated by an experienced professional using the GAS (Gather, Analyze and Summarize) technique, which involves recapping actions (gather), promoting student-centered reflection (analyze), and analyzing lessons learned (summarize)^(19)^. The teledebriefing session lasted for 30 minutes, and all participants were requested to turn on their cameras. Subsequently, the telesimulation evaluation form was administered.

The evaluation form, developed by the author and adapted^(20)^, consisted of 20 items divided into five categories, assessing the objectives, organization, language, appearance, and motivation of nursing students regarding the scenario. The response options were defined as follows: 1 = agree and 2 = disagree, with space provided for comments and suggestions. After analyzing the results, relevant modifications were made, and the final version was presented.

### Data analyze

The data were entered into an Excel® spreadsheet and then exported to IBM® SPSS® Statistics software, version 20.0, for analysis. The Content Validity Index (CVI) was used to calculate the results. The responses “3” and “4” were considered for the calculation of the CVI, indicating agreement (CVI = number of agreements with a score of “3” or “4” / total number of items x 100). Responses rated as “1” or “2” were reviewed^(15)^. The average proportions of the relevant items were calculated (CVI = number of agreements / total number of items x 100). An index equal to or greater than 80% was considered acceptable for the instrument^(21)^. The same procedures and agreement rate were followed for the scenario test. The suggestions provided by the students and experts were transcribed and grouped based on semantic proximity.

## RESULTS

The construction of the telesimulation scenario comprised seven stages: defining learning objectives, creating a clinical case, identifying material and human resources, planning the pre-briefing, structuring the running phase, developing the observer checklist, and planning the debriefing. During the scenario pilot, three students voluntarily participated, with two acting in the scene and one serving as an observer to test the checklist. Adjustments were suggested for the running phase due to delays in image sharing during telesimulation. Consequently, certain image resources, such as glucose results, were removed and could be verbally communicated by the facilitator. No modifications were made to the other aspects of the script.

Regarding content validation, the panel of experts consisted of 10 individuals, with nine (90%) being female and one (10%) being male. All judges (100%) were nurses, with eight (80%) holding a master’s degree and having completed a dissertation in the field. Six judges (60%) had a doctoral degree with a thesis in the field, and eight judges (80%) were specialists in the field. Furthermore, all judges (100%) had published articles in reputable journals. Among the experts, half (50%) had worked as clinical nurses in the field, with the majority possessing 5 to 10 years of experience (30%). Four judges (40%) worked as educators, with two (20%) having 5 to 10 years of experience and two (20%) having over 10 years of experience. Regarding experience with simulation, half (50%) of the professionals had previously utilized simulation in their practice. Only one expert (10%) mentioned having formal training as a simulation instructor and training in telesimulation.

In the evaluation of the 14 scenario items in terms of clarity, three items had the lowest agreement index at 90%, while the remaining 11 items achieved a 100% agreement index. The overall Content Validity Index (CVI) for clarity was 97.8%. Similarly, in terms of relevance, two items had the lowest agreement index at 90%, and the remaining items obtained a 100% agreement index. The overall CVI for relevance was determined to be 98.5%. One expert (10%) identified items 1, 7, and 9 of the scenarios as unclear, and another expert (10%) recommended significant revision for items 9 and 10 of the scenarios (Table 1).

**Table 1:** Experts’ evaluation of clarity and relevance/pertinence of the telesimulation scenario and the CVI of each item, Porto Alegre, Rio Grande do Sul, Brazil, 2022

Items	Clarity CVI (%)	Relevance/Pertinence CVI (%)
1- Telesimulation name	90	100
2- Main learning objective	100	100
3- Secondary objectives	100	100
4- Estimated duration	100	100
5- Required resources and materials	100	100
6- Guidance for actors	100	100
7- Participants	90	100
*8- Pre-briefing guidance*	100	100
9- Scenario description	90	90
10- Clinical case description	100	90
11- Running phase	100	100
12- *Debriefing*	100	100
13- Activity summary by the instructor	100	100
14- Observer checklist	100	100
Overall IVC	97.8%	98.5%

To enhance the scenario, the suggestions provided by the experts were taken into consideration. These suggestions included the following changes: excluding the name and gestational age of the newborn (item 1); replacing “manage care” with “implement care” (item 2); removing the nursing technician and adding another nurse (item 7); including the information “Small for Gestational Age (SGA)” and “from the obstetric center,” reversing the order of “intermediate neonatal care” to “neonatal intermediate care”; replacing “respiratory dysfunction and weight gain” with “prematurity and low birth weight,” as well as replacing “with breastfeeding on demand” with “with oral feeding: breastfeeding on demand”; and including “last fed 3 hours ago, no lacteal supplement received, glucose level at 12 hours of life: 71 mg/dL” (item 10).

In the scenario testing phase, a total of 10 nursing students participated. All participants were above 18 years of age and were either currently enrolled in or had completed the 6th semester of the nursing undergraduate program. Regarding the 20 items on the scenario evaluation form, one item achieved a lower agreement rate of 90%, while the remaining 19 items obtained a 100% agreement rate. The overall content validity index (CVI) was calculated to be 99.7%. The objective, organization, language, and motivation categories received unanimous agreement. Only the third item in the appearance category (“The resources used in the scenario are attractive”) received a slightly lower score (Table 2).

**Table 2:** Evaluation of students regarding the objective, organization, language, appearance, and motivation after testing the telesimulation scenario, Porto Alegre, Rio Grande do Sul, Brazil, 2022

Items	Disagree n (%)	Agree n (%)
Block 1 – Objective		
The telesimulation scenario meets the proposed objectives	-	10 (100)
Assists in experiencing clinical situations	-	10 (100)
The scenario is suitable for use by you at this moment	-	10 (100)
Block 2 – Organization		
The scenario title is appealing	-	10 (100)
The title indicates the content of the scenario	-	10 (100)
The resources used are appropriate	-	10 (100)
The steps of telesimulation have logical sequence	-	10 (100)
There is coherence between the objectives and the content of the telesimulation scenario	-	10 (100)
Block 3 – Language		
The instructions and recommendations for telesimulation are clear and concise	-	10 (100)
The text is interesting	-	10 (100)
The vocabulary used in the scenario is accessible	-	10 (100)
The writing style corresponds to the level of knowledge of the students	-	10 (100)
Block 4 – Appearance		
The telesimulation represents real clinical situations	-	10 (100)
The appearance of the scenario is simple and clear	-	10 (100)
The resources used in the scenario are appealing	1 (10.0)	9 (90,0)
Block 5 – Motivation		
The scenario is suitable for undergraduate students' profile	-	10 (100)
The scenario content is presented logically	-	10 (100)
The resources used in telesimulation promote interaction	-	10 (100)
Telesimulation fosters critical thinking and decision-making	-	10 (100)
Overall CVI	99.7%	

The suggestions from the students regarding the appearance of the scenario were accepted and incorporated into the instrument by the researcher. To preserve the confidentiality of the respondents, the participants’ names were replaced with pseudonyms. They expressed the following:

*My only suggestion would be to invest in more realistic images that depict the actions we performed*. (Student 3)

Additionally, the suggestion to include fifth-semester undergraduate students in the target audience was noted based on the following statement:

*The participants could include students in women’s health and newborn care since it addressed a topic we also learn in that discipline.* (Student 4)

Regarding the objective category of the scenario, the students reported:

*I felt like I was in the unit, it was really good.* (Student 4)

*It was a very enriching experience, it helped me recall and consolidate knowledge, and I’m certain that it positively contributed to my education.* (Student 5)

*Participating in this telesimulation was really beneficial, we truly got an idea of what it’s like in practice*. (Student 10)

The modifications proposed in this study resulted in the final version of the scenario (Chart 1).

**Chart 1:** Telesimulation scenario in the care of late preterm with Hypoglycemia final version, Porto Alegre, Rio Grande do Sul, Brazil, 2022

1 - Telesimulation Name: Late Preterm Newborn (LPTNB) with Hypoglycemia Target Audience: Undergraduate nursing students from the 5th semester.				
2 - Main Learning Objective: To implement nursing care for late preterm newborns with neonatal hypoglycemia, emphasizing knowledge, leadership, and decision-making skills.				
3 - Secondary Objectives: • Perform vital signs assessment and physical examination. • Identify risk factors for neonatal hypoglycemia. • Early recognition of clinical signs of neonatal hypoglycemia associated with risk factors. • Promptly request medical evaluation. • Guide and execute steps of the neonatal hypoglycemia management protocol. • Provide guidance to family members regarding the care provided.				
4 - Duration: • 10 minutes for briefing. • 10 minutes for the running phase. • 30-40 minutes for teledebriefing.				
5 - Material and Human Resources: • Communication application: Microsoft Teams®. • Vital sign simulator: https://sourceforge.net/projects/vitalsignsim/. • Two computers: - Computer 1: Operator control station with an additional screen for extended configuration mode. - Computer 2: Facilitator’s workstation to monitor students’ perspectives. • Background image file: Neonatal inpatient unit. • Image files for projection: Newborn in an incubator with hypotonia and baby being punctured. • Audio file: Newborn crying. • Video file: Newborn sucking maternal breast. • Human resources: One operator, one facilitator, and three actors.				
6 - Guidance for Actors: Doctor: The actor assigned to this role should thoroughly review the case. They will portray a physician in the Neonatal Inpatient Unit and, when prompted, request the necessary infusions as per the protocol. Experienced Nurse (if necessary): If the participant fails to request blood glucose measurement or fails to identify hypoglycemia, the experienced nurse enters the scenario to offer assistance. They provide information but do not remain in the scene. Child’s Mother: Simone, 19 years old. Following a cesarean delivery, she is admitted to the obstetric inpatient unit. She is distressed about being separated from her first daughter. The scenario requires her to seek help from the team, reporting that the baby appears very “soft.” This should challenge the participant in managing the case. If asked, important information to provide includes: “The baby is sleepy while breastfeeding, doesn’t latch well, and I’ve been breastfeeding every 3 hours.”				
7 - Participants: two nurses				
8 - Pre-briefing: • Announce the activity, stating that the scenario will focus on nursing care for late preterm infants. • Provide the reading material in advance. • Explain the steps involved in the telesimulation. • Discuss the adaptation of expectations in the virtual environment. • Present the resources that will be utilized (actors, software, images, videos, audios). • Clarify the communication methods during the scene. • Establish the fictional contract, confidentiality agreement, and the commitment to respect and empathize with colleagues. • Describe the roles of the participants. • Explain the role of observers and the use of the checklist during the scene. • Distribute the papers to the participants as specified in item 7. • Present the clinical case according to item 10.				
9 - Scenario Summary: A 34-week and 4-day-old newborn from the Obstetric Center is admitted to the neonatal inpatient unit due to respiratory dysfunction and weight gain issues. The scene begins with the late preterm newborn in the incubator, accompanied by the mother. The mother informs the team that her daughter appears unwell, mentioning hypotonia and tremors. Participants are expected to promptly identify signs and symptoms of neonatal hypoglycemia, inform the medical team, and implement nursing care for the late preterm newborn with neonatal hypoglycemia, following the protocol and prescription.				
10 - Description of the Clinical Case: The clinical case involves a female newborn named Ana Clara, with a gestational age of 34 weeks and 4 days. She was delivered via cesarean section, weighing 1900 g at birth. Ana Clara is classified as Small for Gestational Age and had APGAR scores of 6 and 8. After being born at the Obstetric Center, she was admitted to the neonatal intermediate care unit at CARE Hospital due to respiratory dysfunction, prematurity, and low birth weight. Currently, she is approaching 24 hours of life and weighs 1850 g. Ana Clara is kept in a heated incubator, receiving ambient air ventilation and has no venous access. She is on an oral diet of breastfeeding on demand, with her last feeding occurring 3 hours ago. She has not received any milk supplement, and her blood glucose level at 12 hours of life was measured at 71 mg/dl. Ana Clara’s mother, Simone, is also admitted to the obstetric inpatient unit and regularly comes to breastfeed her daughter. Upon arrival, Simone noticed that Ana Clara appeared unwell and requested assistance from the nursing team, mentioning that the baby felt “soft” and exhibited tremors. You have been called to manage this case.				
11- *Running* (Scene):				
Time	Monitor settings	Operator actions	Participants’ actions	Clues
0’	Initial state: RR:70mpm HR: 115 bpm AT: 36.6°C Sat: 89%	-Shares monitor screen without data and image of the newborn in the incubator with hypotonia; - When a participant mentions that he has measured vital signs, the data are released on the monitor; - The physical examination data are informed by the facilitator, as requested by the participant.	-Greet and introduce yourself to the mother; -Collect more information about the patient; -Performs the measurement of vital signs; -Performs physical examination of the newborn.	The mother reports that the daughter is very “soft” and with tremors.
2 min	RR: 70mpm HR: 115 bpm AT: 36.6°C Sat: 89%	- The result of the blood glucose measurement is provided verbally by the facilitator (19mg/dl);	-Requests blood glucose measurement by tape; -Identifies hypoglycemia -Requests urgent medical evaluation;	-If the participants do not request the blood glucose dosage. -The most experienced nurse actress enters the scene.
4 min	RR: 70mpm HR: 110 bpm AT: 36.6°C Sat: 88%	- Decreases HR to 110 and Saturation to 88%; -Shares image of punctured newborn and with infusion pump.	- Identifies clinical deterioration. - Performs venipuncture. - Administers a bolus of 10% glucose solution using a infusion pump. - Installs a continuous infusion of 10% glucose solution. - Performs blood glucose measurement using a tape after 30 minutes.	-Doctor enters the scene and requests information, venipuncture and infusions.
6 min	RR: 50mpm HR: 130 bpm AT: 36.6°C Sat: 96%	-Facilitator verbally informs that 30 minutes have passed; -Verbally inform the blood glucose result (75mg/dl) - Change signals according to time settings 6’; -Releases newborn crying sound; - Share video of newborn sucking maternal breast.	-Performs blood glucose measurement and measurement of vital signs; -Guides the team on glycemic control at 1 hour, 2 hours and 4 hours until glycemic stability; - Place the newborn to suck the maternal breast or offer milk formula if the mother is not available; -Instructs the mother on the importance of breastfeeding; -Requests consultation on breastfeeding.	-Mother says: “What a relief that Ana is crying. It must be getting better If the participant does not put the baby to breastfeed, the mother asks if it is possible, as she seems to be hungry. Question if you’re doing it right.
End of scenario				
12- Teledebriefing: Following the conclusion of the scenario, gather all participants, observers, and actors for the debriefing session. Direct the following questions to the participants: How did you feel during the scenario? Can you provide a chronological account of what unfolded in this specific scene? What characteristics are commonly found in late preterm newborns (LPNBs) that were evident in Ana Clara, the newborn in this case? Did Ana Clara possess any risk factors for hypoglycemia? If so, which ones? What symptoms of hypoglycemia did she exhibit? Why was the situation considered urgent? What are the risks associated with hypoglycemia in newborns? According to the protocol, what actions were taken? How important is post-event care? What nursing interventions are necessary for preventing hypoglycemia? What is the relationship between breastfeeding and hypoglycemia? How might LPNB characteristics impact breastfeeding? Was there any behavior missing during the scenario? Is there anything you would have done differently? For the observers, is there anything you would like to add that hasn’t been mentioned by the participants? If you were in an acting role, what would you have done differently? What lessons have been learned from this scenario? Are there any additional areas of knowledge that could be beneficial in this context? Finally, ask the actors: How did you feel portraying the role of [actor/actress role] in this situation? As a concluding question, what was the main takeaway from this experience?				
13- Summary of the Activity by the Instructor: Review the objectives of the telesimulation, referring to items 2 and 3 of the script, and discuss with the larger group whether they were achieved.				
Item 14- Observer *Checklist*	Expected actions	D	ND	PD
Communication	He greeted the family member (the mother) and introduced himself before seeking assistance from the nursing team. He gathered additional information about the patient and urgently requested a medical evaluation. He provided guidance to the family members regarding the nursing care being provided and emphasized the importance of breastfeeding on demand. He discussed the option of using milk from the milk bank if the newborn is drowsy.			
Clinical information	Next, he proceeded to measure vital signs and conduct a physical examination of the newborn			
Identifying signs and symptoms of neonatal hypoglycemia	He addressed the complaint of hypotonia and attended to the concerns of tremors. He analyzed the previous blood glucose results obtained at 2, 4, 6, and 12 hours. He requested a blood glucose measurement at 24 hours of life and identified clinical deterioration based on the following findings: oxygen saturation (Sat) - 89%, respiratory rate (RR) - 70 breaths per minute, heart rate (HR) - 115 beats per minute, and axillary temperature (AT) - 36.6ºC. By evaluating the blood glucose measurement using a tape, he confirmed the presence of hypoglycemia (Result=19mg/dl).			
Management of neonatal hypoglycemia	To address the hypoglycemia, he performed a venipuncture and administered a 3 ml push of 10% glucose solution as per the medical prescription. He provided guidance to nursing technicians on glycemic control at 1 hour, 2 hours, and 4 hours. He initiated a continuous infusion of glucose solution using an infusion pump for 24 hours. He performed a blood glucose measurement 30 minutes after administering the glucose push. He encouraged the newborn to suckle at the maternal breast or offered milk formula if the mother was unavailable. He sought advice on breastfeeding.			

*Notes: NB - newborn; D - done; ND - not done; PD - partially done; RR - respiratory rate; HR - heart rate; Axillary temperature: 36.6 ºC; Oxygen saturation: 89%*

## DISCUSSION

The late preterm newborn (LPTNB) is at a higher risk for metabolic alterations, including hypoglycemia, which can result in severe neurological complications if not identified and treated promptly^(1, 2)^. To minimize potential harm, it is crucial to raise awareness among nursing professionals during their education. The combination of telecommunication resources and simulation provides a means to transfer knowledge and skills learned in the classroom to clinically simulated situations for this purpose.

When constructing the telesimulation scenario, similar steps were described in a validation study focusing on humanized childbirth and delivery^(22)^. However, various references in the literature exist regarding the development of simulation scenarios, and they deserve proper recognition^(10, 11, 23, 24)^. Moreover, some studies have utilized telesimulation without undergoing a content validation process^(8, 25, 26, 27)^.

During the planning of resources, the absence of essential elements such as internet connectivity and audiovisual systems can undermine participants’ engagement and satisfaction with the activity^(25, 27, 28)^. Research emphasizes the need for facilitator training and prior testing of technologies with students to ensure the smooth implementation of scenarios^(9, 25, 26)^. In this study, conducting a pilot of the scenario helped identify issues with image sharing, which were resolved by reducing unnecessary image resources and with the support of a trained operator.

Another aspect to consider is the briefing stage, which involves introducing the environment, the fictional and confidentiality agreements, the participants’ roles, and the presentation of the clinical case^(9)^. Studies indicate that facilitators need to invest time in suspending disbelief, as this feeling is expected in this instructional strategy^(28)^. Therefore, providing explanations about communication during the scene is essential, aligning students’ expectations with the limitations of telesimulation to create a safe learning environment^(8, 26)^.

Furthermore, the involvement of experts in defining learning objectives is emphasized as a crucial element for the scenario, as it aims to assess participants’ performance^(9, 19, 23)^. However, when adapting objectives to virtual environments, it is recommended to consider the type of telesimulation, available resources, and the achievable learning outcomes^(25, 27, 28)^. In this study, the achievement of objectives was determined through participants’ verbalization of actions, which requires knowledge, leadership, and decision-making skills.

Additionally, the prioritization of cognitive (planning/verbalization) and behavioral (attitude/communication) skills over technical skills was emphasized, as indicated in the literature. However, this approach underestimates the impact of online learning. Recent studies have shown improvements in both technical and cognitive domains with the use of telesimulation, but no significant differences in behavioral gains, such as communication skills. The authors suggest that this may be attributed to students’ challenges in communicating effectively in closed loop during remote sessions^(25, 28)^. While there may be limitations in performing technical skills in telesimulation, facilitators can demonstrate and discuss the techniques during the scenario or teledebriefing^(25)^.

In this study, the agreement among the expert committee regarding the items in the constructed scenario was considered adequate in terms of clarity and relevance^(15, 21)^. Content validation contributed to the technical and scientific enhancement of the telesimulation scenario, as observed in similar studies ^(9, 10, 22, 23)^.

During the scenario testing phase, the high level of agreement among nursing academics regarding items such as “met the proposed objectives,” “represents real clinical situations,” and “promoted critical thinking and decision-making” aligns with the findings of another study highlighting the potential of technology to achieve established objectives, recreate real-life clinical situations in a safe teaching environment, and stimulate critical thinking and decision-making^(25, 27)^.

Although there was unanimous agreement that “Telesimulation represents real clinical situations”, there was disagreement regarding the item “The resources used in the scenario are attractive”. In this context, the literature recommends paying attention to physical aspects (use of standardized patients, actors and simulators, images, videos, serious games), conceptual attributes (signs and symptoms consistent with the patient’s diagnosis), and psychological aspects of fidelity (simulator’s voice, actors, team, monitor sound). This involves including stimuli and cues that would typically be present in a real situation^(9, 27)^. In this study, the use of resources that promote engagement and provide real-time feedback on the patient’s clinical situation, such as the virtual vital signs monitor and the actresses who constantly interact with the participants, is highlighted.

Recent literature emphasizes that telesimulation is feasible and well-received from students’ perspectives^(25, 27, 28)^ and more effective than other forms of distance learning^(8, 26)^. However, studies suggest that this teaching strategy may not be as effective as in-person simulation, although it does have the potential to enhance the performance of healthcare students^(8, 25, 27)^.

### Study Limitations

A limitation of the study is the utilization of Fehring’s criteria for expert selection, as it focuses solely on academic environments. Additionally, the scenario testing was conducted with nursing students from a single institution, and it is recommended to expand the study to include participants from other educational institutions.

### Contributions to the Nursing, Health, or Public Policy Field

The implications of this technology for simulation-based education, care, and research in the context of late preterm infant care are significant. It has the potential to enhance the training of nurses in caring for late preterm infants, a population that has historically been overlooked. It can be utilized in both undergraduate and graduate programs. In care settings, it can contribute to evaluating care processes, facilitating the identification of solutions, and translating scientific evidence into safe care practices through in-service training and continuing education. In research, this tool can be employed in various studies, assessing its impact on nursing professionals working in the neonatal field.

## CONCLUSION

The telesimulation scenario developed for the care of late preterm infants with hypoglycemia has undergone validation by experts and testing by nursing students, encompassing essential elements to guide its implementation and ensure reproducibility. Based on student evaluations, the technology successfully met the proposed objectives, facilitated the experience of clinical situations, and stimulated critical thinking and decision-making in a safe learning environment. It is expected that this teaching tool will support educators in conducting telesimulation experiences in undergraduate nursing education and continuing education services. Further research is warranted to assess the impact of the developed scenario on the knowledge and performance of nursing students in caring for late preterm infants.

## Supplementary Material

0034-7167-reben-76-S4-e20220438-suppl01

0034-7167-reben-76-S4-e20220438-suppl02

0034-7167-reben-76-S4-e20220438-suppl03

0034-7167-reben-76-S4-e20220438-suppl04

0034-7167-reben-76-S4-e20220438-suppl05

0034-7167-reben-76-S4-e20220438-suppl06

0034-7167-reben-76-S4-e20220438-suppl07
