# Supplementary material for: Validation of telesimulation in the care of late preterm newborns with hypoglycemia for nursing students
Source: Rev Bras Enferm. 2023 Dec 8;76(Suppl 4):20220438. doi: 10.1590/0034-7167-2022-0438 (PMC10704675; doi:10.1590/0034-7167-2022-0438)
Supplement: 0034-7167-reben-76-S4-e20220438-suppl01 [file 0034-7167-reben-76-s4-e20220438-suppl01.pdf]

CARACTERIZAÇÃO ESTUDANTES DE ENFERMAGEM			
ID	Semestre que está cursando?	Está cursando ou concluiu a disciplina de cuidado em enfermagem na saúde do recém-nascido, criança e adolescente	Voce tem 18 anos ou mais?
E1	6º	SIM	SIM
E2	6º	SIM	SIM
E3	7º	SIM	SIM
E4	6º	SIM	SIM
E5	6º	SIM	SIM
E6	6º	SIM	SIM
E7	6º	SIM	SIM
E8	7º	SIM	SIM
E9	9º	SIM	SIM
E10	6º	SIM	SIM
