# Supplementary material for: Validation of telesimulation in the care of late preterm newborns with hypoglycemia for nursing students
Source: Rev Bras Enferm. 2023 Dec 8;76(Suppl 4):20220438. doi: 10.1590/0034-7167-2022-0438 (PMC10704675; doi:10.1590/0034-7167-2022-0438)
Supplement: 0034-7167-reben-76-S4-e20220438-suppl02 [file 0034-7167-reben-76-s4-e20220438-suppl02.pdf]

ID	Comentários e sugestões - bloco 1	Comentários e sugestões - bloco 2	Comentários e sugestões - bloco 3	Comentários e sugestões - bloco 4	Comentários e sugestões - bloco 5
E1					
E2				Os recursos que se aproximam mais da realidade (exemplo: uso de vídeo ao invés de imagem, simulação de um monitor real com sinais vitais e com som) são imprescindíveis para a experiência, pois evita que seja algo "estático", e consequentemente nos dá a sensação de ser real	A telessimulação propicia muit[illegible] Quando estamos apenas em a[illegible]
E3	Adorei o cenário! Acho que a parte de Sinais Vitais ficou muito bem construída. Minha única sugestão seria em investir em mais imagens realísticas, que retratassem as ações que fazíamos. No caso que participei, por exemplo, poderia ter uma imagem de um braço de RN com acesso venoso e uma da própria infusão em bolus de glicose. Na minha opinião, essa parte em específico do caso, ficou muito no campo do imaginário, pois apenas o monitor dos Sinais era mostrado			Como disse, acho que ainda faltam mais recursos de imagem	

E4	Me senti na unidade, foi muito bom	Acho que podia ter um encerramento melhor. Terminou de forma abrupta e acabamos ficando um pouco confusas			Poderia ter como participantes os aluno de saúde da mulher e do RN, já que tratou de um assunto que aprendemos nessa disciplina também
E5	Foi uma experiência muito enriquecedora, resgatando e consolidando conhecimentos, por este motivo, tenho certeza que contribuiu para minha formação, de manheira positiva				
E6					
E7	Ótima proposta				
E8					
E9					
E10	Foi muito bom participar desta tele simulação, realmente conseguimos ter uma ideia de como é na prática (cravo).	Da forma que foi realizadas a telessimulação pudemos identificar diversos pontos importantes do atendimento ao paciente e ao familiar			
