# Supplementary material for: Validation of telesimulation in the care of late preterm newborns with hypoglycemia for nursing students
Source: Rev Bras Enferm. 2023 Dec 8;76(Suppl 4):20220438. doi: 10.1590/0034-7167-2022-0438 (PMC10704675; doi:10.1590/0034-7167-2022-0438)
Supplement: 0034-7167-reben-76-S4-e20220438-suppl03 [file 0034-7167-reben-76-s4-e20220438-suppl03.pdf]

ID	SEXO	Q1	Q3	Q4	Q5	Q6	Q7	Q8	Q9	Q10	Q11	Q12
J1	1	1	1	1	1	4	0	0	1	0	0	0
J2	2	1	1	0	0	0	0	0	1	2	0	0
J3	1	1	1	1	1	3	0	0	1	3	0	0
J4	1	1	1	1	0	0	1	3	0	0	0	0
J5	1	1	0	1	0	0	1	3	0	0	0	0
J6	1	1	0	1	0	0	1	3	0	0	0	0
J7	1	1	0	0	0	0	1	1	0	0	0	0
J8	1	1	1	1	1	3	1	0	1	3	0	0
J9	1	1	1	1	1	4	0	4	1	2	0	1
J10	1	0	0	1	0	0	0	0	0	0	1	0

LEGENDA

IDENTIFICAГ‡ГѓO DOS JUIZES SEXO CARACTERIZAГ‡ГѓO
iD QUESTГ•ES DESCRIГ‡ГѓO DAS QUESTГ•ES E RESPOSTAS
J1 01- Feminino Q1 "Possui mestrado com dissertaГ§ГЈo na ГЎrea (simulacao materno-infantil ou neo)?"

J2 02- Masculino 00= NГЈo
J3 01= Sim
J4 Q2 "Possui publicacao de artigo indexado na area (simulacao materno-infantil ou neo)"

J5 00= NГЈo
J6 01= Sim
J7 Q3 "Possui doutorado com dissertaГ§ГЈo na ГЎrea (simulacao materno-infantil ou neo)?"

J8 00= NГЈo
J9 01= Sim
J10 Q4 Possui especializaГ§ГЈo na ГЎrea (crianГ§a ou neonatologia)
00= NГЈo
01= Sim
Q5 Atua como Docente na ГЎrea materno-infantil ou neonatal?
00= NГЈo
01= Sim
Q6 tempo como docente
00= nunca atuou
01= menos de 1 ano
02= de 1 a 5 anos
03= 5 a 10 anos
04= mais de 10 anos
Q7 Atua como enfermeiro assistencial?
00= NГЈo
01= Sim
Q8 tempo como enfermeiro assistencial
00= nunca atuou
01= menos de 1 ano
02= de 1 a 5 anos

03= 5 a 10 anos
04= mais de 10 anos

Q9 Utiliza a estratГ©gia de ensino baseado em simulaГ§ГЈo na sua prГЎtica?
00= NГЈo
01= Sim

Q10 tempo que utiliza a simulaГ§ГЈo como estratГ©gia de ensino
00= nunca atuou
01= menos de 1 ano
02= de 1 a 5 anos
03= 5 a 10 anos
04= mais de 10 anos

Q11 Possui formaГ§ГЈo como instrutor de simulaГ§ГЈo?
00= NГЈo
01= Sim

Q12 tem capacitacao em telessimulaГ§ГЈo
00= NГЈo
01= Sim
01= Sim
