# Supplementary material for: Validation of telesimulation in the care of late preterm newborns with hypoglycemia for nursing students
Source: Rev Bras Enferm. 2023 Dec 8;76(Suppl 4):20220438. doi: 10.1590/0034-7167-2022-0438 (PMC10704675; doi:10.1590/0034-7167-2022-0438)
Supplement: 0034-7167-reben-76-S4-e20220438-suppl04 [file 0034-7167-reben-76-s4-e20220438-suppl04.pdf]

ID	1.1	1.2	1.3	2.1	2.2	2.3	2.4	2.5	3.1	3.2	3.3	3.4	4.1	4.2	4.3	5.1	5.2	5.3	5.4
E1	1	1	1	1	1	1	1	1	1	1	1	1	1	1	1	1	1	1	1
E2	1	1	1	1	1	1	1	1	1	1	1	1	1	1	1	1	1	1	1
E3	1	1	1	1	1	1	1	1	1	1	1	1	1	1	0	1	1	1	1
E4	1	1	1	1	1	1	1	1	1	1	1	1	1	1	1	1	1	1	1
E5	1	1	1	1	1	1	1	1	1	1	1	1	1	1	1	1	1	1	1
E6	1	1	1	1	1	1	1	1	1	1	1	1	1	1	1	1	1	1	1
E7	1	1	1	1	1	1	1	1	1	1	1	1	1	1	1	1	1	1	1
E8	1	1	1	1	1	1	1	1	1	1	1	1	1	1	1	1	1	1	1
E9	1	1	1	1	1	1	1	1	1	1	1	1	1	1	1	1	1	1	1
E10	1	1	1	1	1	1	1	1	1	1	1	1	1	1	1	1	1	1	1

LEGENDA:

IDENTIFICAГ‡ГѓO	ITENS	DESCRIГ‡ГѓO	RESPOSTAS
ID		Bloco 1-objetivo	1=CONCORDO
E1	1.1	O cenГЎrio de telessimulaГ§ГЈo atende aos objetivos propostos	0= NГѓO CONCORDO
E2	1.2	Auxilia na vivГЄncia de situaГ§Гµes clГnicas	
E3	1.3	O cenГЎrio estГЎ adequado para ser usado por vocГЄs nesse momento	
E4		Bloco 2 -organizaГ§ГЈo	
E5	2.1	O tГtulo do cenГЎrio Г© atrativo	
E6	2.2	O tГtulo indica o conteГєdo do cenГЎrio	
E7	2.3	O recursos utilizados estГЈo adequados	
E8	2.4	Os passos da telessimulaГ§ГЈo tГЄm sequГЄncia lГіgica	
E9	2.5	HГЎ coerГЄncia entre os objetivos e o conteГєdo do cenГЎrio de telessimulaГ§ГЈo	
E10		Bloco 3- Linguagem	
	3.1	As orientaГ§Гµes e recomendaГ§Гµes para a telessimulaГ§ГЈo estГЈo claras e objetivas	
	3.2	O texto Г© interessante	
	3.3	O vocabulГЎrio utilizado no cenГЎrio Г© acessГvel	
	3.4	O estilo da redaГ§ГЈo corresponde ao nГvel de conhecimento dos discentes	
		Bloco 4- Aparencia	
	4.1	A telessimulaГ§ГЈo representa situaГ§Гµes clГnicas reais	
	4.2	A aparГЄncia do cenГЎrio estГЎ simples e clara	
	4.3	O recursos utilizados no cenГЎrio sГЈo atrativos	

Bloco 5- MotivaГ§ГЈo

5.1 O cenГЎrio Г© apropriado para o perfil dos discentes de graduaГ§ГЈo

5.2 O conteГєdo do cenГЎrio se apresenta de forma lГіgica

5.3 A recursos utilizados na telessimulaГ§ГЈo promovem a interaГ§ГЈo

5.4 "A telessimulaГ§ГЈo propicia a pensamento crГtico e tomada de decisГЈo"
