# Supplementary material for: Validation of telesimulation in the care of late preterm newborns with hypoglycemia for nursing students
Source: Rev Bras Enferm. 2023 Dec 8;76(Suppl 4):20220438. doi: 10.1590/0034-7167-2022-0438 (PMC10704675; doi:10.1590/0034-7167-2022-0438)
Supplement: 0034-7167-reben-76-S4-e20220438-suppl05 [file 0034-7167-reben-76-s4-e20220438-suppl05.pdf]

ID	CLAR1	CLAR2	CLAR3	CLAR4	CLAR5	CLAR6	CLAR7	CLAR8	CLAR9	CLAR10	CLAR11	CLAR12	CLAR13	CLAR14
J1	4	4	4	4	3	4	3	4	4	4	4	4	4	4
J2	4	4	4	4	4	4	4	3	4	3	4	3	4	4
J3	4	4	4	4	4	4	4	4	4	4	4	4	4	4
J4	2	4	4	4	4	3	4	3	3	3	3	4	4	3
J5	4	4	4	4	4	4	2	4	4	4	4	4	4	4
J6	4	4	4	4	3	4	3	4	4	4	3	4	4	4
J7	4	4	4	3	4	4	4	4	4	3	4	4	4	4
J8	4	3	4	4	4	4	3	3	3	4	3	3	3	3
J9	3	3	3	4	3	3	3	3	2	3	3	4	4	4
J10	3	4	4	4	4	4	4	4	4	4	3	4	4	3

LEGENDA

CLAREZA
CLAR 1 A 14   01= nΓJo clara

02= pouco clara

03= bastante clara

04= muito clara
