# Supplementary material for: Validation of telesimulation in the care of late preterm newborns with hypoglycemia for nursing students
Source: Rev Bras Enferm. 2023 Dec 8;76(Suppl 4):20220438. doi: 10.1590/0034-7167-2022-0438 (PMC10704675; doi:10.1590/0034-7167-2022-0438)
Supplement: 0034-7167-reben-76-S4-e20220438-suppl06 [file 0034-7167-reben-76-s4-e20220438-suppl06.pdf]

ID	RELEV1	RELEV2	RELEV3	RELEV4	RELEV5	RELEV6	RELEV7	RELEV8	RELEV9	RELEV10	RELEV11	RELEV12	RELEV13	RELEV14
J1	4	4	4	4	3	4	4	3	4	4	4	4	4	4
J2	4	4	4	4	4	4	4	3	4	2	4	3	4	4
J3	3	4	3	4	4	4	4	4	4	4	4	4	4	4
J4	3	4	4	4	4	4	4	4	4	3	4	4	4	4
J5	4	4	4	4	4	4	4	4	4	4	4	4	4	4
J6	4	4	4	4	4	4	3	4	4	4	3	4	4	4
J7	4	4	4	4	4	4	4	4	4	3	4	4	4	4
J8	4	3	4	4	4	4	4	4	4	4	4	4	4	4
J9	3	3	3	4	3	3	3	3	2	3	3	4	4	4
J10	3	4	4	4	4	4	4	4	4	4	3	4	4	4

LEGENDA

RELEVΓ,NCIA

RELEV 1 A14 01= nΓJo relevante ou nΓJo representativo

02= item necessita de grande revisΓJo para ser representativo

03= item necessita de pequena revisΓJo para ser representativo

04= item relevante ou representativo
