# Supplementary material for: Validation of telesimulation in the care of late preterm newborns with hypoglycemia for nursing students
Source: Rev Bras Enferm. 2023 Dec 8;76(Suppl 4):20220438. doi: 10.1590/0034-7167-2022-0438 (PMC10704675; doi:10.1590/0034-7167-2022-0438)
Supplement: 0034-7167-reben-76-S4-e20220438-suppl07 [file 0034-7167-reben-76-s4-e20220438-suppl07.pdf]

ITENS DO ROTEIRO	CONSIDERAÇÕES DOS JUIZES
**Item 1 - Nome da Telessimulação: Recém-nascido Pré-termo Tardio (RNPT-T) com Hipoglicemia Neonatal, Ana Clara, Idade Gestacional: 34 semanas e 4 dias, público-alvo: graduandos em enfermagem a partir do 5º período.**	J1- Existe a necessidade do título ser tão longo? Precisa ter o objetivo e público alvo? Não poderia ser apenas: Hipoglicemia em RNPTT. J3-Na minha opinião o título contém informações que não são relevantes (nome e idade gestacional). J4- Excluir nome do neonato e IG do título e apresentá-lo entre aspas. Sugestão: “Recém-nascido Pré-termo Tardio (RNPT-T) com Hipoglicemia Neonatal.” J6-Nome da RN precisa estar no título da telessimulação? J8- Título adequado. Porém ao apresentar a atividade aos estudantes deverá ser modificado, afinal anuncia o objetivo de intervenção ao qual o cenário se destina. J9-Título extenso. Sugiro: Manejo do recém-nascido pré-termo tardio com hipoglicemia neonatal. Grau de clareza: bastante e muito claro são semelhantes, sugiro substituir bastante claro por claro, apenas. J10- Sugiro: Recém-nascido Pré-termo Tardio (RNPT-T), 34 semanas e 4 dias, Ana Clara, Hipoglicemia Neonatal, público-alvo: graduandos em enfermagem a partir do 5º período.
**Item 2- Objetivo principal de aprendizagem:**<br>**● Gerenciar o atendimento de enfermagem ao RNPT-T com hipoglicemia neonatal, por meio do conhecimento, liderança e tomada de decisão;**	J5- - “Compreender e gerenciar o …” - “Atendimento” ou “Cuidado”? J8- Utilizando a taxonomia de bloom sugiro o termo “implementar” ou “aplicar”. Assim ficaria: Implementar ou Aplicar o atendimento de enfermagem ao RNPT-T com hipoglicemia neonatal, por meio do conhecimento da liderança e tomada de decisão baseada em evidências.<br>J9- Substituir “Gerenciar o atendimento ... por, “Gerenciar o cuidado de enfermagem ao RNPT-T com...” Santos JLG, et al. Práticas de enfermeiros na gerência do cuidado em enfermagem e saúde: revisão integrativa. Rev Bras Enferm, Brasília 2013 mar-abr; 66(2): 257-63.
**Item 3 - Objetivos secundários:**<br>**● Solicitar mensuração dos sinais vitais;**<br>**● Solicitar dados do exame físico;**<br>**● Solicitar coleta de hemoglicoteste;**<br>**● Identificar precocemente os sinais e sintomas de hipoglicemia neonatal;**<br>**● Solicitar preparo e administração de medicamentos;**<br>**● Manejar a hipoglicemia neonatal conforme protocolo (Atenção à saúde do recém-nascido : guia para os profissionais de saúde, Volume III – Problemas respiratórios, cardiocirculatórios, metabólicos, neurológicos, ortopédicos e dermatológicos);**<br>**● Monitorar os resultados das intervenções de enfermagem;**<br>**● Refletir sobre o papel do enfermeiro no atendimento ao RNPT-T com hipoglicemia neonatal, como integrante da equipe multidisciplinar;**	J3- Talvez seja interessante incluir um objetivo sobre passar as informações sobre a saúde do RN à mãe. J4- Objetivos propostos estão adequados. J5- - Seria adequado também acrescentar “Solicitar avaliação médica com urgência” ou algo do gênero? Para introduzir o personagem no cenário e também para obter a prescrição. - E também algum feedback/orientação à família (comunicação, segurança). J8- Ao fazer a leitura pensei que neste cenário o enfermeiro (estudantes que estão em aprendizagem neste cenário) poderiam realizar a avaliação dos sinais e exame físico. Assim modificaria os dois primeiros objetivos secundários para “realizar”. O último objetivo secundário sugiro o termo “avaliar” a conduta do enfermeiro, pois assim eles poderão refletir e pensar sobre. J9- Sugiro<br>- Identificar fatores de risco para hipoglicemia neonatal<br>- Identificar sinais clínicos de hipoglicemia (relato materno, dados de exame físico e registros de sinais vitais<br>- Orientar e executar etapas do protocolo de manejo da hipoglicemia neonatal. (Coletar HGT por fita. Interpretar HGT por fita. Avaliar condições para VO. Gerenciar o preparo de material para procedimento de punção vascular. Executar punção vascular. Administrar infusão rápida de glicose. Gerenciar preparo da infusão EV SG10%).J7-Sugiro alterar o trecho: Identificar precocemente os sinais e sintomas de hipoglicemia neonatal, RELACIONANDO AOS FATORES DE RISCO.
**Item 4- Tempo de duração previsto: 10 min (Briefing), 15 min (Running), 30-40 min (Debriefing);**	J4- Tempos de briefing, execução e reflexão adequados.
**Item 5- Recursos e Materiais necessários:● Aplicativo de comunicação Microsoft Teams® (Teams Login | Acessar plataforma colaborativa Microsoft Teams): aplicativo para videoconferência licenciado pela UFRGS nas atividades educacionais remotas. Este espaço educacional virtual permite a interação de alunos em grupos de estudos, salas de aula virtual e interações professor-aluno em tempo real. Possibilita o compartilhamento de tela, configuração de discussões em modo juntos, para que todos apareçam de forma digital na mesma tela facilitando as interações síncronas. ● Software Vital Sign (https://sourceforge.net/projects/vitalsignsim/): software gratuito que simula um monitor simples de sinais vitais. Permite o controle e alteração dos sinais vitais através do teclado do computador. ● 2 computadores: (computador 1- estação de controle do operador com tela extra para configuração no modo estender) e (computador 2 -para monitorar a visão dos alunos); ● Imagem da unidade de internação neonatal para aplicar como plano de fundo dos atores; ● Imagens que serão projetadas: imagem do RNPT-T na incubadora com hipotonia; ● Som: som de recém-nascido chorando; ● Recursos humanos: 1 operador; 1 facilitador e 3 atores;**	J1- Não compreendi se é necessário todos instalarem o software vital sign. J6- Havia ficado em dúvida quem são: operador, facilitador e atores, mas na sequência fica claro o papel de cada um. Em relação aos 2 computadores, um é para fazer as alterações e outro para observar os alunos, isso? J9- Substituir: Imagem por Arquivos de imagem e Som por Arquivos de áudio. Recursos humanos: Atividade dirigida à formação de enfermeiros. Considerando o objetivo da telessimulação, a técnica de enfermagem deve ser atriz no contexto do cenário.
**Item 6- Orientação aos Atores:**<br>**● Médico(a): Este ator precisa revisar cuidadosamente o caso. Assumirá o papel de médica da Unidade de internação neonatal, quando chamada irá solicitar as infusões conforme protocolo.**<br>**● Enfermeiro (a) mais experiente (se for necessário): caso o acadêmico não solicite a dosagem da glicemia e não identifique a enfermeira mais experiente entra no cenário oferecendo ajuda. A mesma fornece informações, mas não permanece no cenário.**<br>**● Mãe da criança: A mãe, Simone, tem 19 anos. Após o parto cesáreo é admitida na unidade de internação obstétrica. Está aflita por ter que ficar longe da sua primeira filha. O cenário requer que a mãe solicite ajuda da equipe, referindo que a bebê está muito “molinha”. É necessário instigar o participante no manejo do caso. Informações importantes que devem ser relatadas aos participantes caso seja questionada: sonolento para mamar, não pega bem a mama, vem de 3/3 horas amamentar.**	J9- Acrescentar orientações à técnica de enfermagem.
**Item 7- Participantes: ● Enfermeiro- 01 ● Técnico de enfermagem-01**	J1- Não compreendi sobre os participantes não incluirem os atores. J5- - Os alunos farão o cenário em dupla, um sendo Enf e outro Téc, alternando? Perdão se não entendi bem. J6- Fiquei na dúvida, pois nas orientações temos 3 atores (médico, enfermeiro experiente caso o acadêmico não solicite o hemoglicoteste e mãe da criança), mas na parte de participantes temos só enfermeiro e técnico de enfermagem. J9- Sugiro: Acadêmica 1 representa uma enfermeira<br>Acadêmica 2, representa outra enfermeira.
Item 8- Briefing ● Explicar a atividade de telessimulação; ● Explicar sobre a importância da confidencialidade e sobre o contrato de ficção; Dicas para os observadores da telessimulação: ○ Ouvir atentamente, relacionando o tema com os conteúdos teóricos; ○ Respeitar os colegas que estão encenando, evitando críticas desnecessárias; ○ Falar somente quando for solicitado pelo professor, sugerindo construtivamente. ● Distribuir os papéis para os participantes ● Iniciar a descrição do caso.	J2- Sugiro informar no roteiro como será a escolha dos participantes e como será a distribuição dos papeis. J4- Sugestão: talvez descrever brevemente sobre o contrato de ficção adotado. J9- Sugiro: “Explicar a atividade de telessimulação, informando sobre o ambiente, equipamentos e cenário. Deslocar, “Dicas para os observadores...” para o final do item 8.

**Item 9- Descrição do cenário: Recém-nascida (RN) com 34 semanas e 4 dias foi admitida na unidade de internação neonatal após o nascimento. A RNPT-T inicia com sinais e sintomas de hipoglicemia neonatal, a mãe da criança solicita ajuda. Os participantes irão avaliar a paciente e manejar a situação conforme protocolo. A paciente irá apresentar hipotonia, taquipnéia, choro fraco e cianose perioral e tremores. Espera-se que os participantes identifiquem precocemente os sinais e sintomas de hipoglicemia neonatal e iniciem o tratamento conforme protocolo.**	J5- - "…iniciem o tratamento - manejo?". J7- -Sugiro alterar o trecho: Espera-se que os participantes identifiquem precocemente os sinais e sintomas de hipoglicemia neonatal, comuniquem a equipe médica e iniciem o tratamento conforme protocolo e prescrição. J9- Citar procedência do RNPT-T, centro obst. ou aloj. Conjunto. Descrever cenário quanto ao leito do RN na UINeonatal, monitores, incubadora ... A frase A RNPT-T inicia com sinais e sintomas de hipoglicemia neonatal, a mãe da criança solicita ajuda, associa o diagnóstico de hipoglicemia neonatal à mãe.
**Item 10- Descrição do caso clínico: Recém-nascido (RN) de sexo feminino, Ana Clara, com idade gestacional de 34 semanas e 4 dias, parto cesáreo, peso ao nascer 1900g, APGAR 6 e 8. Foi admitida na Unidade de cuidados neonatais intermediários do Hospital Care devido à disfunção respiratória e para ganho de peso. No dia de hoje está completando 24 horas de vida, com peso atual 1850kg. Mantida em incubadora, com aleitamento materno por livre demanda, sem acesso venoso. Mãe de Ana Clara (Simone) está internada na Unidade de internação obstétrica (UIO) e vem regularmente amamentar sua filha. Ao chegar na unidade, solicitou ajuda pois achou a bebê muito "molinha" e com tremores. Você é enfermeira da unidade e é chamada para atender este caso. Histórico de saúde/Dados clínicos/Exames:**<br>**Revisão dos sistemas (objetivo): Geral: Sonolento, cianose perioral, hipotônico, taquipneico e com tremores.**<br>**Peso atual: 1.850kg**<br>**Respiratório: Som respiratório normal, taquipnéia Cardiovascular: som cardíaco normal , ritmo regular.**<br>**Gastrointestinal e**<br>**urinário: sem anormalidades**<br>**Neurológico: sonolento**<br>**pele: palidez, cianose perioral**<br>**musculoesquelético: hipotônico**<br>**alimentação: aleitamento materno por livre demanda, sucção débil.**<br>**Exames:**<br>**Dosagem da Glicemia: 2h= 65mg/dl, 4h= 70mg/dl, 6h= 67 mg/dl, 12 h= 71 mg/dl**	J2- Sugiro alterar o título do item. "Passagem de plantão" condiz melhor com as informações presentes no item. A redação confunde com o item 9. J4- Sugestão: RN internou em UTI Neo devido disfunção respiratória e ganho de peso. Na coleta dos dados clínicos/exame, não é abordado dentro das condições ventilatórias do RN se o mesmo necessita de aporte de oxigenoterapia ou não (ventila em ar ambiente espontaneamente ou com aporte de oxigênio por CEN? Oxigênio em incubadora? etc). Sugiro especificar, mesmo que o foco seja a hipoglicemia. J5- - Bem sucinto, gostei. J7- -Sugiro adicionar ao menos mais um fator de risco relevante para desenvolvimento de hipoglicemia do RN; a prematuridade por si só é um fator de risco, porém, para exercitar o raciocinio clínico, seria importante adicionar, por exemplo no histórico: DMG materna, assim como, RN PIG. Assim, incentivamos o olhar critíco para a população de risco, visando a prevenção de desfecho desvaforável. - Sugiro fornecer a informação de quanto tempo se passou desde que o RN sugou o seio materno (ou recebeu complemento, por exemplo) até o momento em que apresenta sintomas, tendo em vista que este é um dado essencial e sempre questionado inicialmente pela equipe de enfermagem e médica, nesse tipo de avaliação a beira de leito. -Sugiro alterar o seguinte trecho: Mantida em incubadora aquecida, com dieta por via oral: aleitamento materno exclusivo por livre demanda. J9- Acrescentar dados relevantes ao caso clínico: Motivo da internação (disfunção respiratória, prematuridade, baixo peso). - Estado nutricional ao nascer;<br>valor do 1ºHGT; FR desde o nascimento; SatO2 desde nascimento; FC desde nascimento; perfusão periférica; TAx desde o nascimento; Eliminações fecais e urinárias.
**Item 11-Running (cena):**	J1- Adequar no 6 minuto que teoricamente está estabilizado a temperatura para 36,5 pois 36,4 é hipotermia. J4- Running muito bem contruído. J5- - Talvez acrescentar, só pra ficar bem claro, que o que acontece no 6' é 30min após a infusão de glicose. J6- A solicitação da verificação da glicemia capilar poderia ser no minuto 0, após exame físico alterado (hipotonia e tremores). J7- Sugiro adicionar ao final a oferta do seio materno, preconizado pelo protocolo, ou oferta de fórmula láctea por via oral, conforme prescrição, caso o RN não apresente prontidão para mamar ou a mãe não esteja presente ao final do atendimento para ofertar o seio. J9- O caso clínico aponta valores normais de HGT até a 12ª h de vida. Entendi que a idade do RNPT-T na etapa zero minuto da cena seria de 24h; assim, o HGT da 24ª h de vida já deveria ter sido feito? Incluir orientações sobre cuidado materno ao RNPT-T. J10- No item 7 (Participantes) consta 1 enfermeiro e 1 técnico de enfermagem porém não ficou clara as funções durante a realização da cena.
**Item 12- Debriefing: Questionar os PARTICIPANTES: ● Como você se sentiu? ● O que aconteceu nesta cena? ● Conforme o protocolo de atendimento, o que foi feito? ● Como foi controlar a situação? ● O que você faria diferente? ● O que pensou enquanto atuava na cena? ● A situação vivenciada aqui tem relação com o mundo real? Questionar os OBSERVADORES: ● O que aconteceu nesta cena? ● O que você faria diferente? ● Quais foram os aprendizados do cenário? ● Que outros conhecimentos poderiam ajudar neste atendimento? Questionamentos finais: ● O que vocês aprenderam com a situação vivida? ● De que forma essa experiência ajudará na prática profissional?**	J2- Quem serão os participantes do debriefing? Somente os alunos que participaram ou os atores poderão contribuir com a discussão?
**Item 13- Resumo da atividade pelo professor: ● Rever os objetivos da telessimulação: ler os objetivos e verificar em grupo se estes foram alcançados.**	J4- Sugestão: Seria interessante o professor retomar os cuidados de enfermagem pós-evento (hipoglicemia) em virtude do RNPT-T ser população de risco para tal. Além disso, um detalhe importante é o neonato apresentar hipotermia (36,4ºC) ao final da cena devido o manuseio– outro evento que contribuí para hipoglicemias recorrentes, além das dificuldades de amamentação. Nesse sentido, talvez seja pertinente e relevante abordar as características específicas do RNPT-T na discussão (além do protocolo do MS para manejo de hipoglicemia).
**Item 14- Checklist para observadores:**	J5- - Acrescentar a solicitação da avaliação médica. J10- As ações esperadas são somente do participante enfermeiro? E no caso do participante técnico de enfermagem realizar a ação esperada? Como vai ser sinalizado na coluna de observação?
